# Nitrate Ingestion: A Review of the Health and Physical Performance Effects

**DOI:** 10.3390/nu6115224

**Published:** 2014-11-18

**Authors:** William T. Clements, Sang-Rok Lee, Richard J. Bloomer

**Affiliations:** Cardiorespiratory/Metabolic Laboratory, Department of Health and Sport Sciences, University of Memphis, 106 Roane Fieldhouse, Memphis, TN 38152, USA; E-Mails: tclements1228@gmail.com (W.T.C.); srlee1@memphis.edu (S.-R.L.)

**Keywords:** nitrate, nitric oxide, exercise, blood flow, beetroot, blood pressure

## Abstract

This paper provides an overview of the current literature and scientific evidence surrounding inorganic nitrate (NO_3_^−^) supplementation and its potential for improving human health and physical performance. As indicative of the ever-expanding organic and natural food consumer market, athletes and health enthusiasts alike are constantly searching for ingredient-specific “super foods” and dietary supplements capable of eliciting health and performance benefits. Evidence suggests that NO_3_^−^ is the viable active component within beetroot juice (BRJ) and other vegetables, responsible for health-promoting and ergogenic effects. Indeed, multiple studies support NO_3_^−^ supplementation as an effective method to improve exercise performance. NO_3_^−^ supplementation (either as BRJ or sodium nitrate [NaNO_3_^−^]) has also demonstrated modest benefits pertaining to cardiovascular health, such as reducing blood pressure (BP), enhancing blood flow, and elevating the driving pressure of O_2_ in the microcirculation to areas of hypoxia or exercising tissue. These findings are important to cardiovascular medicine/exercise physiology and suggest a possible role for NO_3_^−^ supplementation: (1) as a low-cost prevention and treatment intervention for patients suffering from blood flow disorders; and (2) an effective, natural ergogenic aid for athletes. Benefits have been noted following a single bolus, as well as daily supplementation of NO_3_^−^. While results are promising, additional research is needed to determine the impact of NO_3_^−^ supplementation on anaerobic exercise performance, to identify principle relationships between isolated nitrate and other ingredients found in nitrate-rich vegetables (e.g., vitamin C, polyphenols, fatty acids, thiocyanate), to explore the specific dose-response relationships needed to elicit health and ergogenic benefits, to prolong the supplementation period beyond a relatively short period (*i.e.*, >15 days), to determine if more robust effects can be observed with longer-term treatment, and to fully examine the safety of chronic NO_3_^−^ supplementation, as this continues to be a concern of some.

## 1. Introduction

Daily lifestyle behaviors such as dietary intake, physical activity, and sleep patterns are perhaps the strongest influencing factors related to human health and athletic performance. Hence, much of the ongoing research is focused on the impact of specific lifestyle factors, including the selective use of isolated nutrients and nutritional supplements to enhance human health and functional capacity. Indeed, health enthusiasts and athletes alike are constantly searching for key nutritional ingredients and associated “super foods” that may enhance the structure and function of the body, maintain health, thwart the onset of chronic disease, and increase longevity. Indicative of increased consumer interest, the Nutrition Business Journal (NBJ) noted (from 1999 to 2009) that the US market for organic and natural foods experienced an 8.6% increase in annual growth [1]. The steadfast dietary supplement market continues to maintain pace, with NBJ appraisals of $32 billion in sales for 2012, up 7% from the prior year [1]. Current estimates indicate that the natural and organic juice market is also on the rise, with growth of 13% in 2012, yielding an astounding $2.4 billion in sales from fresh and canned artifacts [1]. Selective nutrition for health and performance outcomes is not only trending, but arguably the focal point for today’s consumers.

Consider the common beetroot (BR) (*Beta vulgaris*), known to be a rich source of antioxidants and micronutrients including potassium, betaine, sodium, magnesium, vitamin C and perhaps most notably, inorganic nitrate (NO_3_^−^). The inorganic anion is found in generous amounts in green leafy vegetables, and also substantially found in a variety of vegetable and fruit juices including: beetroot juice (BRJ), V8^® ^(CSC Brands, LP, Camden, NJ, USA), carrot juice and pomegranate juice [2]. NO_3_^−^ has received considerable attention in recent years and is quickly gaining traction as a health and performance enhancing nutritional supplement. NO_3_^−^-rich BRJ has also recently gained popularity for proposed anti-cancer and anti-inflammatory properties, and a reduced risk for adverse cardiovascular outcomes including stroke, myocardial infarction, systemic and pulmonary hypertension, as well as the formation of gastric ulcers (Detopoulou *et al.* [3], Lundberg *et al.* 2008 [4]).

Once ingested, inorganic NO_3_^−^ metabolizes *in vivo* to bioactive nitrite (NO_2_^−^) and is subsequently salvaged and circulated in human blood [4]. Interestingly, bacteria located in the oral cavity are integral in bioactivating and reducing NO_3_^−^ to NO_2_^−^, through an enzymatic reduction process now commonly referred to as the enterosalivary pathway [4,5,6]. Indeed, NO_2_^−^ anion is now widely recognized as a transient species that critically functions in the physiological regulation of blood flow and blood pressure [7,8]. NO_2_^−^ exerts these effects in the body via its conversion to functional nitrogen oxides (NO_x_), including nitric oxide (NO) [4]. NO is a diffusible free radical molecule with multiple biological functions—one of which is to signal smooth muscle within the inner lining (endothelium) of blood vessels to relax, subsequently enhancing blood flow.

Hence, the theory proposed by many companies who market “NO-stimulating supplements” is that by elevating NO, the flow of oxygenated blood to working skeletal muscle tissue may be enhanced, ultimately aiding exercise performance and recovery. To this effect, several molecules (e.g., l-arginine, carnitine, 2-nitrooxy ethyl 2-amino 3-methylbutanoate, arginine-alpha-ketoglutarate) have been marketed to the bodybuilding community as NO boosters, with proposed health and performance enhancing benefits. However, when administered orally, these molecules have only demonstrated modest evidence for elevating NO levels, with either little or no evidence of a performance improvement following supplementation. To the contrary, mounting evidence now exists surrounding the benefits of NO_3_^−^ supplementation on exercise performance outcomes.

Hoon and associates recently examined the effect of NO_3_^−^ supplementation on exercise performance in humans [9]. The groups’ meta-analysis examined 17 studies that utilized NO_3_^−^, of which BRJ and sodium nitrate (NaNO_3_^−^) were most commonly supplemented. Supplemental doses generally ranged from 300 to 600 mg and were administered as either a single bolus or up to 15 days of consistent consumption. Upon interpretation, the group showed a noteworthy modest benefit (ES = 0.79, 95% CI: 0.23–1.35) of NO_3_^−^ administration on exercise performance for time to exhaustion tests (*p* = 0.006). The analysis also demonstrated a minor yet statistically insignificant favorable outcome on performance for time trials (ES = 0.11, 95% CI: −0.16–0.37) and graded exercise performance tests (ES = 0.26, 95% CI: −0.10–0.62). The groups’ analysis also proposed that the benefits to athletic performance were mostly seen in individuals who were inactive to recreationally active for constant load time to exhaustion performances [9]. In spite of these findings, the minor beneficial effect may be significant in an elite competitive sport setting [10], although this remains unresolved.

It has recently been reported that dietary NO_3_^−^, administered in the form of BRJ or NaNO_3_^−^, enhances exercise tolerance, reduces blood pressure (BP), and lowers the oxygen (O_2_) cost of cardiovascular exercise [11,12,13,14,15]. Several studies indicate that NO_3_^−^, through conversion to NO, is the active component within BR and other dark green vegetables responsible for regulation of BP [16,17,18,19,20,21,22,23], blood flow [16,24,25,26,27], gastric integrity [4,16,21], and the extent of tissue protection following ischemic insult [16,18,26]. The NO_3_^−^ can be delivered either as a component of food—typically about 500 mL or 2 cups of BRJ [9,11,12,19,28,29,30,31,32,33,34,35,36,37], whole BR [38] or as NaNO_3_^−^—typically about 10 mg/kg of body mass [15,39,40,41]. Dietary NO_3_^−^ supplementation has also been shown to reduce the ATP/PCr cost associated with force production, thereby enhancing muscle contractile efficiency [11], in addition to improving the efficiency of oxidative phosphorylation [14].

Historically, NO was thought to solely be produced endogenously via the oxidation of l-arginine by nitric oxide synthases (NOS). NO_3_^−^ and NO_2_^−^ were believed to be mere inert metabolic end-products of this “linear” l-arginine/NO pathway. However, it is now widely accepted that food and supplement sources of NO_3_^−^ act as exogenous donors of NO_2_^−^, NO, and other NO_x_. The recognition of this reversible step-wise reduction mechanism (NO_3_^−^**→**NO_2_^−^**→**NO**→**NO_x_) is profound, predominately due to nitrite’s capacity to be reduced in blood and tissue under certain physiological conditions [7,8,24,42,43,44,45]. Specifically, conditions of low O_2_ tension (e.g., ischemic and hypoxic tissue) and pH [46] in various body regions markedly promote reduction of NO_2_^−^ to NO. These human physiological conditions are characterized by a largely deoxygenated venous circulation, and effectively maximize nitrite’s potency as a vasodilator in the systemic circulation, thereby producing NO in body regions of most need. Because these conditions may persist in active, exercising skeletal muscle, diet manipulation via NO_3_^−^-rich sources may also provide an effective means to enhance blood flow during exercise. Hypoxic vasodilation is of particular interest to investigators and practitioners as it is a fundamental physiological process that links the flow of blood and oxygen to its demand in body tissues—a process that could potentially be utilized in both athletic and diseased populations.

In 2011, Lansley and colleagues demonstrated that 6 day supplementation (500 mL/day, ~6.2 mmol of NO_3_^−^) of NO_3_^−^-rich BRJ in humans reduced resting systolic blood pressure (SBP) and pulmonary O_2_ consumption (V̇O_2_) during walking and running exercise [13]. The authors used ion exchange resins to selectively remove NO_3_^−^ from the BRJ (500 mL/day, ~0.003 mmol of NO_3_^−^). In doing so, they created a placebo juice, free of NO_3_^−^, yet identical in terms of color, taste, smell, and texture. This allowed for a targeted analysis of NO_3_^−^ in regards to its effect on blood pressure and the physiological responses to exercise. The selective removal of NO_3_^−^ from BRJ resulted in no changes to plasma NO_2_^−^ concentration, blood pressure, or the physiological responses to exercise [13]. These findings suggest that inorganic NO_3_^−^ anion is likely an essential nutrient found in vegetable rich diets. Moreover, these findings indicate that the reductions to SBP and V̇O_2_ are principally attributable to the high NO_3_^−^ content of BRJ (>250 mg/100 g of fresh weight) and not the other constituents [13].

In some cases, supplementation has shown to improve one’s tolerance to exercise [30,37,47]. These studies effectively demonstrate a health and performance-enhancing role for dietary NO_3_^−^ supplementation. Particularly, NO_3_^−^ ingestion may prove viable as an adjunct therapeutic agent for treatment and prevention of hypertension, and more generally “blood-oxygen” delivery disorders commonly associated with metabolic syndrome and the onset of chronic disease [4]. As such, several nutritional supplements containing NO_3_^−^ have recently been introduced to the consumer market, claiming various improvements in health and exercise performance.

Considering the increasing interest in NO_3_^−^ supplementation, the purpose of this review is to provide an up to date summary of research pertaining to dietary NO_3_^−^ supplementation for purposes of enhancing human health and performance. This review includes studies involving both normal environments and those of hypoxic conditions.

## 2. History

Initially described by Roy and Brown in 1879, hypoxic vasodilation is a preserved physiological reaction to inadequate regional or gross body O_2_ supply [48]. Effectively, it is the body’s means of coupling blood flow and, hence, O_2_ distributions to tissue metabolic demand. Hypoxic vasodilation is dependent upon a detection mechanism whereby specific feedback vasodilator operators identify discrepancies between tissue O_2_ consumption and “real-time” oxygenated blood delivery [49]. The specific elementary reactions inherent to this O_2_ sensing mechanism and many of the identities of key vasodilator effectors went mostly undetected for several years [7].

Dating back to 1953, however, Furchgott and Bhadrakhom [50] demonstrated nitrite’s ability to vasodilate pre-constricted rabbit aortic vessels. Even in the 1970s and early 1980s, Murad’s group and Ignarro’s group mirrored these earlier findings; upholding the demonstrated vasodilatory potential of NO_2_^−^ in aortic rings via activation of soluble guanylyl cyclase [51,52,53]. These pioneering scientists utilized micromolar to millimolar concentrations of NO_2_^−^ to achieve these vasodilatory effects *in vitro*. It was also shown in 1994 by two groups independently that NO is generated in the stomach from acidified NO_2_^−^ [42,43]. The NO generated in the acidic environment of the stomach is around 10–100 ppm and is more than sufficient, actually several magnitudes higher than the limiting threshold necessary to induce vasodilation. However, due to the fact that its vasodilatory properties were only exhibited at concentrations several orders of magnitude higher than that seen physiologically, the interest in nitrite’s vasodilatory potential was limited.

Lauer and colleagues demonstrated in 2001 that NO_2_^−^ exhibited no capacity to vasodilate when infused into human forearms at concentrations of 200 μmol/L [54]. Hence, given the nanomolar concentrations present in mammalian blood, NO_2_^−^ was negated as an effective blood vessel dilator as late as the early 2000’s [54,55,56]. In spite of apparent NO_2_^−^ inactivity, Gladwin and colleagues identified an arterial-venous gradient in NO_2_^−^ which markedly increased consumption of NO_2_^−^ during exercise, in which the NOS pathway is hindered in humans [7]. These findings led the group to propose a now broadly accepted concept—NO_2_^−^ is a likely precursor of NO, preferentially reduced to NO in anoxic and acidic environments [4,46].

Modin and colleagues demonstrated that NO_2_^−^ functioned to dilate aortic ring preparations at physiological concentrations when the pH in the medium mirrored ischemic and metabolically active tissues [46]. In 2003, Cosby and associates showed that infused NO_2_^−^ in extremely minor micromolar concentrations significantly improved blood flow in human circulation—an effect that was pronounced when subjects underwent exercise performance [24]. Hence, the past 15 years have proved extremely monumental as it is now apparent that blood and tissue NO_2_^−^ is reduced *in vivo* to form NO and modify blood flow [7,24].

Recent studies performed by Ferguson and colleagues [57,58] provide insight into the role of beetroot juice to improve metabolic control. For example, one study focused on skeletal muscle hindlimb (composed of 28 muscles and muscle parts) blood flow and vascular conductance in 19 adult male Sprague-Dawley rats during submaximal treadmill running [58]. At random, rats were assigned to consume either tap water or BR-infused water for a 5 day period with a NO_3_^−^ dose of approximately 1 mmol/kg/day. The BR was diluted in 100 mL of tap water and the rat consumption was observed on a daily basis to ensure plasma NO_3_^−^ and NO_2_^−^ levels corresponded to those seen in human studies. Compared to the control group, the BR group showed greater elevations in total exercising skeletal muscle blood flow and vascular conductance. Of note, the NO_3_^−^ supplemented group demonstrated enhanced blood flow and vascular conductance to primarily fast-twitch type II + d/x muscle fibers [58]. However, for the first time, despite a ~10 mmHg reduction in mean arterial pressure (MAP), changes in exercising skeletal muscle blood flow were positively augmented. These findings confirm the role of NO_3_^−^ in the regulation of muscle O_2_ perfusion and are likely the result of its reduction to NO_2_^−^ and subsequent reduction to NO *in vivo*. Moreover, these findings suggest that increased blood flow and hence increased O_2_ delivery may sufficiently increase the driving pressure of O_2_ across the capillary-myocyte interface [58].

These improvements to blood flow and vascular conductance to skeletal muscle were observed without any decreases to the splanchnic circulation. Hence, the authors proposed that the enhanced skeletal muscle blood flow and vascular conductance were likely due to improved cardiac output and stroke volume rather than blood redistribution effects from vasoconstriction mechanisms of the splanchnic circulation. These data provide evidence that NO_3_^−^ ingestion increases muscle O_2_ delivery in a fiber-dependent manner post-reduction to NO_2_^−^ and NO.

To this effect, it is apparent why modulating the NO_3_^−^**→**NO_2_^−^**→**NO via the diet may prove beneficial for physiologists, dieticians, nutritionists, and physicians alike.

## 3. What is Nitrate?

Nitrate (NO_3_^−^) is an inorganic polyatomic anion that exists naturally in the environment. It is present in both air and drinking water, as well as in certain foods, and is produced endogenously from a family of nitric oxide synthases (NOS) via oxidation of the amino acid l-arginine. Nitrate is found primarily in the diet (>80%) as an inorganic component of vegetables [4]. Predominate sources of nitrates are beets, celery, lettuce, radishes, and spinach [59] and are rather prevalent in green vegetables produced in heated glasshouses or hydroponically grown [60]. NO_3_^−^, through conversion to nitrite (NO_2_^−^), is also a common food preservative found with bacon, bologna, hot dogs, and luncheon meats [61]. Nitrite consumption is mainly found exogenously via consumption of cereal products, vegetables, and cured meats [61,62]. NO_3_^−^ and NO_2_^−^ have been used for centuries to maintain flavor and color in processed and cured meats [16], and have demonstrated anti-microbial [63] and anti-fungal properties [64]. In the form of potassium NO_3_ (saltpeter), the anion is in fact a natural impurity of salt, and historically contributed to the pinkish-red hue observed in salted meats [16].

Following oral consumption, NO_3_^−^ is quickly absorbed in the duodenum and jejunum and is dispersed amongst the whole body [65]. Post-absorption, NO_3_^−^ begins circulation within plasma and has a half-life of about 5 h. Following uptake from the blood, NO_3_^−^ is concentrated in saliva via an active transport system. The NO_3_^−^ is concentrated by at least 10-fold in saliva and is capable of reaching concentrations up to several millimolar [66]. The explanation for why NO_3_^−^ is concentrated in human saliva remains largely uncertain [16], but it is estimated that up to 25% of dietary NO_3_^−^ is salvaged in this enterosalivary circulation [16]. The larger portion is excreted via the kidneys. Once secreted in the oral cavity, commensal bacterial anaerobes—located predominantly in the crypts of the tongue—bioactivate NO_3_^−^ and reduce it to NO_2_^−^ in saliva. Roughly 20% of this salivary NO_3_^−^ is reduced, which translates to approximately 4%–8% of the ingested NO_3_^−^ dose [66,67,68]. Using NO_3_^−^-reductase enzymes, these oral microfloras use NO_3_^−^ as an alternate one electron acceptor and reduce it to NO_2_^−^ [5].

Once swallowed, salivary NO_2_^−^ has demonstrated ability to convert to NO in the acidic stomach [42,43], but it is also evident that some of this NO_2_^−^ is absorbed to increase circulating plasma NO_2_^−^ concentrations [4]. Many tissues and enzymatic systems of the body have been described to reduce NO_2_^−^; a number of hemeproteins (e.g., myoglobin, hemoglobin, xanthine oxidase) also procure capacity to reduce NO_2_^−^ [69]. Additionally, it is also known that oxygenated myoglobin and hemoglobin effectively seek out NO and oxidize it to NO_3_^−^. It has been estimated that NO yield rates may be as fast as one to tens of nanomolar per second under physiological conditions [69]. Additionally, it is apparent that some NO_2_^−^ conversion to NO is found in the gut and is likely due to its innate diverse characteristics (e.g., large latitudes of pH and oxygen partial pressures, gastric content). For a detailed review of the mechanisms of action of nitrate metabolism, refer to [10,69].

Hyde and colleagues recently measured the NO_3_^−^ reducing capacity of tongue-scraping samples from 6 healthy volunteers, and analyzed the metagenomes of the microbial communities to identify bacteria that predominately contribute to NO_3_^−^ reduction in the oral cavity. The group found a number of bacterial genera that significantly contributed to reduction of NO_3_ˉ including: *Veillonella*, *Neisseria*, *Haemophilus*, *Porphyromonas*, *Fusobacterium*, *Prevotella* and *Leptotrichia* [6]. Secondly, utilizing a whole genome shotgun sequencing approach, the group also identified 14 *candidate species that significantly contributed to* NO_3_^−^ reduction. Interestingly, the groups’ use of a community approach allowed for specific identification of bacteria that may directly or indirectly influence NO_3_^−^ bioactivation in the oral cavity. Fundamental questions still remain as to whether or not specific bacterial communities correlate to NO insufficiency and an inflated risk for cardiovascular disease. *In this regard, multi-biofilm analyses and larger subject groups are needed to truly elucidate specific bacteria integral to* NO_3_^−^ bioactivation as this will likely reveal stronger correlations between oral health and systemic disease [6]. Additionally, it is likely that oral bacterial communities differ amongst different races, patient populations, and between individuals in different geographical locations [6]. If this is indeed so, enhancement of bacterial communities specific for NO_3_^−^ reduction in patient populations may prove beneficial.

Further, the use of certain mouthwashes may be urged against if such bacterial treatments are used in the future. This is due to the fact that chlorhexadine-based mouthwashes have shown to inhibit response to NO_3_^−^ and actually heighten BP values in both animal and human studies, implicating the necessity of oral bacteria for NO_3_^−^ bioactivation [18,21]. It is still unclear whether alcohol-based mouthwashes and chlorinated swimming pools exert similar negative BP effects as those seen in chlorhexidine-based mouthwash studies; perhaps these may also be considered in the foreseeable future [6].

In order to determine the amount of ingested NO_2_^−^ that actually reached that reaches the systemic circulation in humans, Hunault and others [70] supplemented 9 volunteers with single sodium NO_2_^−^ doses equal to approximately 150 and 300 mg (0.06 mmol NaNO_2_ and 0.12 mmol NaNO_2_/mmol of Hb, respectively). The volunteers were given NaNO_2_^−^ in aqueous form, in an effort to most closely mirror what happens physiologically, as the majority of human exposure to NO_2_^−^ is a result of salivary reduction of NO_3_^−^ [62]. The authors concluded that the absolute bioavailability of NO_2_^−^ (*i.e.*, the amount that reached the systemic circulation) was 95% and 98% respectively for the two doses. These findings suggested to the group that NO_2_^−^ is almost completely absorbed in the duodenum and jejunum and that its first pass effect through the liver is minimal at best [70].

## 4. Importance of Nitric Oxide

Before discovery in the 1980s of endogenous NO biosynthesis, it was inconceivable that cells would purposely produce a potentially toxic molecule like NO, also known as endothelium-derived relaxing factor. The colorless and odorless gas is considered a reactive nitrogen species and is a common pollutant in air. It is also a component in cigarette smoke, and a toxic gas found in the exhaust of cars and jet planes known to generate acid rain and eradicate the ozone [16]. However, the picture is now clear that NO plays numerous biological roles when available at physiological concentrations, functioning and affecting neurotransmission, wound healing, tumors, asthma, glaucoma, hemoglobin delivery of oxygen, blood flow, thrombosis (*i.e.*, blood clotting), muscle contractility, differentiation and proliferation of stem cells, glucose and calcium homeostasis, and mitochondrial O_2_ consumption, while also being associated with learning and memory formation [17,25,71]. The gas is also a principal neurotransmitter that mediates inflammation, host defense, and even penile erectile function [16]. It should be noted that this list represents a small fraction of the many biological processes that are inherent to NO. Though the molecule possesses a very simple molecular structure, the breadth of NO involvement in the human body remains a topic of continued investigation.

NO is primarily of interest to investigators and practitioners due to its potential for relaxing human vasculature [16]—an effect that may result in improved blood flow during rest [27] and exercise [27,72]. Vasodilation is paramount to human health, as the process itself is utilized by the body to deliver oxygenated blood to areas of the body that are in greatest need (e.g., contracting skeletal muscle, beating hearts, ischemic areas, *etc*.). Vasodilation is not solely used for delivery of oxygenated blood, but also for the delivery of glucose, lipids, and other nutrients to a variety of tissues.

Given the wide variety of biological processes known to be regulated by NO (mainly the ability of NO to augment blood flow), it is apparent why maintaining a continuous circulating pool of NO might be beneficial to both athletes and those who suffer from ischemia and hypoxia-related conditions. Because NO is a gas, produced endogenously from the amino acid l-arginine—by three isoforms of NOS, it is very difficult to provide oral supplementation of NO directly (Note: oral supplementation with l-arginine is also largely ineffective for elevating circulating NO). However, through consumption of dietary supplements and certain donor drugs, as well as through intake of vegetables rich in NO_3_^−^, clinicians and exercise scientists have evaluated alternative methods to increase circulating NO, independent of its endogenous NOS biosynthesis.

As a therapeutic agent and NO donor, inorganic nitrate and nitrite act to dilate blood vessels. However, analysis of the pharmacodynamic profiles between organic and inorganic nitrate show striking differences for inducing vasodilation in blood vessels, particularly in regards to potency. Inherent to pharmaceutical agents like nitroglycerine and isosorbide mononitrate (e.g., organic nitrate) is their capacity to act as lipophilic dilators, converting rapidly to NO in smooth muscle via the mitochondrial enzyme, aldehyde dehydrogenase [69]. Inorganic nitrate and nitrite also actively convert to NO and induce vasodilation, but pharmacodynamically exert their dilatory effect at much lower orders of magnitude. Organic nitrate is frequently used in the cardiovascular field of medicine and can be administered via several forms (e.g., tablet, transdermal patch, ointment, in solution for intravenous purposes, as an aerosol spray delivered sublingually) [16]. However, it is well known in the medicinal field that nitrovasodilators such as nitroglycerin are subject to tolerance in human patients and may, over time, decrease their effectiveness for promoting vasodilation [16].

In this regard, inorganic nitrate and nitrite may prove rather advantageous as possible contenders for future treatment and prevention of ischemia-related illnesses. Additionally, the conversion from inorganic NO_2_^−^ to NO occurs along a pH and pO_2_ gradient (e.g., acidic and oxygen-deficient environments [46]. At such high concentrations, NO and its reactions products are toxic to many enteropathogens (e.g., *Salmonella*, *Shigella*, *Helicobacter pylori*) and principle mediators of host defense [73]. Many enzymes possess the capacity to reduce NO_2_^−^ to NO, however none may be as important as the enzymes innate to the hemeproteins myoglobin (tissue) and deoxyhemoglobin (plasma) along the oxygen gradient. NO_2_^−^ likely reacts with myoglobin and deoxyhemoglobin via a similar mechanism where its reduction to NO is enhanced in anoxic and acidic conditions [7]. NO functions to dilate arterioles and allow for more O_2_-carrying-Hb to reach hypoxic and ischemic tissue beds [51,52]. The pH and pO_2_ gradient is of paramount importance as NO_2_^−^ has been shown to enhance blood flow selectively in anoxic environments and may allow for blood to flow to areas where it is most necessary [7,58,71].

Nitrate from dietary sources may be paramount in the future of cardiovascular medicine by allowing generation of NO to localized areas experiencing hypoxia (e.g., oxygen deficiency) and ischemia (blood deficiency). Hypothetically, inorganic nitrate may match NO to areas most needed rather than globally making NO available throughout the body and running the risk of hypotension from whole-body vasodilation. Perhaps most intriguing is that the amount of inorganic NO_3_^−^ to effectively manipulate the NO_3_^−^**→**NO_2_^−^**→**NO pathway and promote the NO-pool can be obtained via a diet rich in vegetables [4,16].

Aside from its vasodilatory potential, NO acts to decrease myointimal hyperplasia [74,75,76]. This condition is characterized by an abnormal narrowing of arteries resulting from proliferation and migration of vascular smooth muscle cells as well as expansion of extracellular matrix in the intima of affected arteries. NO plays a key role in this pathophysiological process by inhibiting adherence and infiltration of monocytes and can even signal apoptosis of affected smooth muscle cells. Furthermore, an increase in circulating levels of NO_2_^−^/NO may protect hypoxic tissues (e.g., damaged blood vessels, local ischemic areas, contracting skeletal muscle) by improving blood and O_2_ perfusion to ischemic areas. The increase in blood flow would theoretically stimulate O_2_ and nutrient delivery (e.g., amino acids and glucose) to exercising skeletal muscle, hence aiding exercise performance [28]. Several mechanisms have been proposed for this NO_2_^−^-derived NO matching of blood flow to areas experiencing O_2_ deprivation [28,55,77,78]. Due to this significant capacity for improving human circulatory system, NO-stimulating therapeutics and NO_3_^−^ supplements are novel targets for cardiovascular health investigators, clinicians, and sport supplement companies alike [4,8].

## 5. Nitrate and Health

As reported by the World Health Organization, cardiovascular disease accounts for 40% of all deaths. Astoundingly, in 2006, total health care costs in the US were upwards of $2 trillion dollars (~$7000 dollars/person) and expected to double by 2015 according to the National Healthcare Statistics Group. The costs alone associated with chronic “lifestyle” diseases that include obesity, diabetes, hypertension, and coronary artery disease are responsible for 75% of the nation’s annual cost of health care [16]. Many of these chronic diseases involve a malfunctioning endothelium and an inability to adequately produce and maintain NO homeostasis [16]. In this regard, a new trend has seemed to emerge in the nutrition arena, suggesting that along with reduced caloric and fat intake, diets rich in food that stimulate NO bioavailability (e.g., NO_3_^−^ and NO_2_^−^-enriched foods) are viable options for seeking a variety of health benefits. Indeed, along with daily physical activity, dietary intake should be considered a first-line target for disease prevention [16].

In the United States, dietary intake of NO_3_^−^ has been estimated to be about 40 to 100 and 31–185 mg/day, with approximately 85% coming solely from vegetables in the diet [59,79]. This intake can be greatly enhanced to reach more than 1200 mg (~20 mmol) by including high NO_3_^−^ foods, as a part of the Dietary Approaches to Stop Hypertension (DASH) diet. Interesting to note, this intake is approximately 5-fold higher than the World Health Organization’s Acceptable Daily Intake (ADI) of 3.7 mg of NO_3_^−^ per kg of body weight per day and more than a 2-fold higher than the United States Environmental Protection Agency of 7.0 mg of NO_3_^−^ per kg of body weight per day [2]. Some DASH studies have proposed that the diet may be as efficacious as any single hypotensive agent in regards to reducing BP [80].

Perhaps most intriguing, however, is that nearly identical (and sometimes even greater) reductions have been seen with moderate doses of dietary NO_3_^−^ [20,23]—although much more research is needed to definitively state this [16]. Indeed, NO_3_^−^ is likely the major component of vegetable-rich diets and likely one of many responsible for the beneficial responses to BP. Yet, until recently, the anion has remained largely ignored. However, it is apparent that it is an essential nutrient inherent to vegetable and fruit-rich diets such as Mediterranean and traditional Japanese diets, that have long been affiliated with protection against cardiovascular disease and diabetes mellitus type 2 [16].

### 5.1. Toxicity of NO_3_^−^ and NO_2_^−^

Historically, NO_3_^−^ and NO_2_^−^ have also been considered to be toxic to humans by causing the formation of N-nitrosamines [68], which are potential carcinogens [81,82,83]. It has also been formerly stated that inorganic sources of NO_3_^−^ and NO_2_^−^ from the diet are critical in development of cancer including gastric [67] and bladder cancer [82]. Due to these findings, several FDA restrictions were put in place in past years to regulate NO_3_^−^ and NO_2_^−^ levels in both food and drinking water within the US.

However, current critical reviews of NO_3_^−^ in animal toxicology literature indicate no evidence for carcinogenesis or mutagenesis [16,61]. Furthermore, current epidemiological data do not provide strong evidence supporting restriction of dietary NO_3_^−^ and are intrinsically unsubstantial [16]. It has recently been shown that NO_2_^−^, at concentrations (e.g., 0.5–10 mmol/L) that mimic human saliva, possesses both cytocidal and cytostatic anti-microbial properties for six common oral pathogens *in vitro* [84]. Although some evidence refutes this, the anti-microbial effect of NO_2_^−^ has also been demonstrated to be more pronounced with increasing acidity of the culture medium (Xia *et al.* [84]). At very low concentrations (e.g., 0.2 mM) and in pH environments below 5.0, NO_2_^−^ may also modulate growth and survival of bacteria associated with periodontal disease [63]. Also of interest is that consumption of NO_3_^−^ and NO_2_^−^ along with antioxidants (significant components in fruits and vegetables) may inhibit the formation of nitrosamines in the gastric milieu [16].

Human studies have shown an association between elevated levels of NO_3_^−^ exposure and certain cancers but their correlations are not fully established [16]. In 2006, the International Agency for Research on Cancer (IARC) reviewed carcinogenic risk along with NO_3_^−^ and NO_2_^−^ consumption. The IARC concluded that food and drinking water sources of NO_3_^−^ and NO_2_^−^ were not strongly supported by epidemiological evidence [85]. The IARC did, however, conclude that endogenous nitrosation and gastric cancer were associated normally under conditions of low vitamin C intake and high NO_2_^−^ levels (commonly found in cured meats) [85]. However, vitamin C is found in many fruit and vegetable sources and is particularly high in BRJ. It is evident that longstanding concerns about the toxicity of orally consumed NO_3_^−^ and NO_2_^−^ (specifically those from natural vegetable sources) have been overstated [16].

### 5.2. Health Benefits of NO_3_^−^ and NO_2_^−^: Blood Pressure

The health promoting effects of NO_3_^−^ have been rigorously investigated over the past several years. In addition to NO_3_^−^, BRJ also contains many antioxidants that may possess protective properties associated with exercise-induced oxidative stress [86]. BRJ has also recently gained popularity for proposed anti-cancer and anti-inflammatory properties, and a reduced risk for adverse cardiovascular outcomes including stroke, myocardial infarction, systemic and pulmonary hypertension, as well as the formation of gastric ulcers [3,4,87]. Despite all of these inherent qualities, NO_3_^−^ research is relatively new in terms of supplementation in humans.

In fact, it was not until 2006 that NO_3_^−^ was first orally supplemented by Larsen and colleagues. They tested 17 healthy, normotensive subjects using a double blind crossover study to assess the several effects of NO_3_^−^ including reduction in blood pressure [20]. Subjects consumed doses (0.1 mmol kg^−1^day^−1^) of either NaNO_3_ or placebo sodium chloride (NaCl) for two, 3 day periods separated by a 10 day washout period. Both the NO_3_^−^ and placebo were dissolved in water and were indistinguishable by taste or appearance. The dosage corresponded to an intake of 150–250 g of NO_3_^−^-enriched vegetables such as spinach, lettuce, or beetroot. They noted average reductions in DBP of 3.7 mmHg following NO_3_^−^ consumption compared to placebo [20]. MAP was also lower by approximately 3.2 mmHg, however, SBP remained unchanged. Their findings along with others were one of the first to demonstrate that oral NO_3_^−^ likely led to formation of the potent vasodilator NO, *in vivo*.

In support of these findings, Webb and associates also tested the effects of oral NO_3_^−^ administration and its effects on BP and vasoprotection [23]. 14 healthy subjects drank 500 mL BRJ or water. The group then measured BP every 15 min for 1 h prior to ingestion and 3 h post-ingestion—then hourly to 6 h with a final BP reading at the 24 h mark. The authors noted peak reductions in SBP (~10.4 mmHg, *p* < 0.01) and DBP (~8.1 mmHg, *p* < 0.01) within 2.5 and 3 h post-consumption, respectively. BP changes also remained for up to 5 h post-ingestion. The reductions in BP began 1 h following consumption of BRJ compared to water, with the BP reductions corresponding with the peak increases in plasma concentrations of NO_2_^−^. Plasma concentrations of NO_2_^−^ also peaked 3 h post-ingestion and remained elevated for approximately 5 h following administration of the BRJ.

The group also demonstrated cytoprotective properties of NO_3_^−^-rich BRJ [23]. Indeed, the group showed BRJ’s capacity for reversal of endothelial dysfunction associated with ischemia reperfusion injury in the brachial artery. Utilizing adenosine di-phosphate (ADP) and collagen stimuli, they were also able to demonstrate *ex vivo* inhibition of platelet aggregation following BRJ administration. These findings effectively demonstrated that NO_3_^−^ is a major component of vegetable rich diets responsible for cytoprotection and reductions to BP. In a co-study, the group also demonstrated that the changes to BP and plasma NO_2_^−^ concentrations were obliterated if the subjects spit out their saliva [23].

A year later, Petersson and collegaues were able to demonstrate that the gastroprotective and BP reducing responses to oral doses of NO_3_^−^ were negated following use of antiseptic mouthwash in mice [21]. These findings significantly corroborated the likelihood of an enterosalivary pathway and most striking—the host-symbiotic relationship between humans and oral bacteria in regards to the bioactivation and hence, positive health benefits associated with ingestion of NO_3_^−^.

In opposition to the above findings, Murphy and colleagues failed to note a decrease in SBP or DBP in response to BR ingestion [88]. However, the authors concluded that this was likely due to the blood pressure measurements taken within 1 h of whole BR ingestion. Given the above findings by Webb *et al*. [23], it is apparent that peak reductions in BP are directly proportional to peak plasma NO_2_^−^ concentrations, which appear 2.5 to 3 h marker following BR ingestion. Since these studies, several have others have also noted significant consistent reductions to BP.

In 2010, Kapil and colleagues [18] similarly showed that ingestion of potassium NO_3_^−^ capsules resulted in plasma NO_2_^−^ concentrations that were both dose and time dependent. Interestingly, the group also noted sex differences in response to NO_3_^−^ supplementation. The group found that the rises in plasma NO_2_^−^ and plasma NO_3_^−^ following potassium NO_3_^−^ capsule ingestion were significantly lower in males than in females. However, the fold increases were relatively similar with an approximately 3 fold increase in males and a 4 fold increase in females. Contrarily, men demonstrated greater responses to potassium NO_3_^−^ in terms of BP. Both SBP and DBP were attenuated more significantly in males than in females. The group proposed that the differences in responses to NO_3_^−^ supplementation may be due to incident lingual bacterial species differences amongst men and women, and possibly different *in vivo* handling of NO_x_ [18]. More research is currently needed in this area to identify definitive sex differences in response to NO_3_^−^ ingestion. 

In another study in 2013, Ghosh and colleagues [89] studied the effects of increased systemic plasma NO_2_^−^ levels in 15 drug naïve grade 1 hypertensives. Using dietary NO_3_^−^ doses of approximately 3.5 mmol, the group found that NO_2_^−^ levels were elevated by ~1.5 fold. The dose caused substantial reduction to SBP (~12 mmHg). They effectively demonstrated that NO_3_^−^ supplementation was indeed an effective, low-cost approach to the treatment of hypertension.

In 2013, Wylie and others investigated BRJ and the pharmacodynamics dose-response relationships in 10 healthy men [90]. Using a balanced crossover study design, the group administered doses of 70, 140, 280 mL of concentrated BRJ (4.2, 8.4 and 16.8 mmol NO_3_^−^, respectively) or no supplement to investigate the responses on resting plasma [NO_3_^−^] and [NO_2_^−^] over a 24 h period. The group mirrored Webb’s findings [23]—noting dose-dependent increases in plasma [NO_3_^−^] and [NO_2_^−^] and peak plasma [NO_2_^−^] at the 2–2.5 h marker—post-ingestion of the 4.2 and 8.4 mmol NO_3_^−^ doses. The group also noted lagging peak increases in plasma [NO_2_^−^] juxtaposed with plasma [NO_3_^−^]. These findings further reflect the reduction of NO_3_^−^ by lingual bacteria in the enterosalivary circulation. They also found that peak plasma [NO_2_^−^] for the 16.8 mmol dose occurred approximately 3 h post ingestion. Given the above findings by Webb and Wylie, it is apparent that peak reductions in BP are directly proportional to peak plasma NO_2_^−^ concentrations, which appear 2.5 to 3 h marker following BR ingestion. Since these studies, several have others have also noted significant consistent reductions to BP.

### 5.3. Health Benefits of NO_3_^−^ and NO_2_^−^: Blood Flow and Associated Parameters

As previously stated, it has been reported that diets consisting of NO_3_^−^-rich foods are associated with inhibition of platelet aggregation and preservation and improvement of endothelial dysfunction [16,23,71]. Given that aging in healthy males is associated with endothelial dysfunction [91] and lower exercising blood flow [91,92,93,94], it may be particularly important for older men to maintain NO_2_^−^/NO pools in the case of endogenous NOS disturbance [4].

BRJ and NO-stimulating supplements provide a means to enhance NO_2_^−^ and NO concentrations and ensure these concentrations are maintained in the event of endogenous NOS disturbance [95]. In animal studies, NO has proven capacity to decrease leukocyte and platelet adhesion to endothelial walls [71,75] as well as decrease proliferation of smooth muscle cells [74,75,76]. NO_3_^−^ may also correlate to reductions in arterial stiffness, inflammation, and intimal thickness [96]. These findings are indicative of an emerging consensus that manipulation of the NO_3_^−^→NO_2_^−^→NO pathway may prove meaningful in a chronic disease context [4,16].

Inability to sufficiently produce NO is also known to be an early event in the formation of atherosclerotic lesions and is associated with several risk factors for cardiovascular disease, including peripheral artery disease (PAD) [91]. PAD is a form of cardiovascular disease caused by atherosclerotic occlusions that significantly arrest blood flow to the lower extremities. PAD has been reported to affect approximately 27 million people in Europe and North America [97]. About one-third of all patients with PAD suffer from discontinuous claudication that occurs during walking and improves with rest [98]. Claudication results directly from and inability to match the O_2_ demand of exercising tissue. In this regard, many cardiovascular and metabolic disorders (e.g., hypertension, atherosclerosis, diabetes mellitus, hypercholeresterolemia), including PAD, are developed in the presence of NO deficiency and oxidative stress [16].

Recently, Kenjale and colleagues [37] supplemented 4 men and 4 women (age 67 ± 13) suffering from intermittent claudication with inorganic NO_3_^−^ in the form of BRJ or PL (500 mL). BRJ raised plasma levels of NO_2_^−^ specifically at the 3 h post-ingestion marker, which was associated with decreased BP during rest and exercise, increased claudication onset time (~18%), and peak walking time (~17%). Subjects who ingested BRJ also had lower SBP and heart rate (HR) values at 2 min into the recovery period. This suggests NO_2_^−^-related NO signaling increases blood flow to the extremities in areas of tissue hypoxia and thus increases exercise tolerance in subjects with PAD. The authors also utilized near-infrared spectroscopy (NIRS) to analyze gastrocnemius (calf) muscle tissue oxygenation in response to a maximal cardiopulmonary exercise test (CPX) and found increased oxygenation in specific indices of the tissue [37]. These results further validate vasodilation and perfusion in peripheral tissue following BRJ consumption.

Another recent animal study was performed by Hendgen-Cotta and associates documented the benefits of NO_3_^−^ enriched water supplementation (NaNO_3_) in the improvement of revascularization in mice induced with chronic ischemia [26]. The authors also found, when administering a commercially available antibacterial (*i.e.*, chlorhexidine-containing) mouthwash daily for 1 week to NO_3_^−^ ingesting mice, that the conversion of NO_3_^−^ to NO_2_^−^ in the oral cavity was significantly suppressed. Consequently, the BP-lowering effects and gastroprotective effects of NO_3_^−^ were diminished. These results suggest that excessive use of commercially available antiseptic mouthwashes may significantly reduce the bioactivity of NO_3_^−^ [21,26]. Govoni and colleagues also demonstrated that plasma NO_2_^−^ levels are markedly decreased following NO_3_^−^ ingestion when individuals also use antiseptic mouthwash [99]. Consumers looking for NO_3_^−^ benefits from BRJ and other vegetables should likely avoid chronic use of commercially available antibacterial (e.g., chlorohexidine-containing) mouthwash [99]. In a similar manner, it should be noted that the chlorine in swimming pools may also alter oral microflora activity in the human mouth due to the common contact of the mouth with water while swimming [39], which may reduce plasma NO_2_^−^ significantly [26]. Bescos and others have also proposed that disinfectants present in chlorinated pools may attenuate microbial reductase activity; although this is an area that requires further research [39]. Considering the above, investigators who are conducting research trials involving NO_3_^−^ may attempt to control for both the use of antiseptic mouthwash and the use of chlorinated swimming pools by research participants.

## 6. Nitrate Supplementation and Exercise Performance

 A recent study was performed by Hoon and colleagues [100] that tested NO_3_^−^ supplementation in national-level cyclists and their response to high-intensity exercise. Using two cycle ergometer tests with each lasting 4 min, 26 cyclists performed the test followed by a 75 min rest interval. Intriguingly, the authors utilized a “multi-dose” approach—in which each cyclist performed the exercise test under all 4 separate dosing regimens. The separate conditions consisted of: 70 mL of NO_3_^−^-rich BRJ 150 min or 75 min before the initial cycling time trial, and an additional “booster-dose” of 35 mL in the 150 min condition post-first trial and a placebo. Although the mean findings in the 3 NO_3_^−^ groups were inconclusive for the first trial, the authors found that the subsequent cycling trial was potentially harmful. They also noted a lack of consistency within individuals. These findings suggest that NO_3_^−^ may be particularly ineffective and perhaps even detrimental to high-intensity cycling exercise those performing at a competitive level.

Indeed, Many authors have now reported that NO_3_^−^ supplementation (mainly administered via BRJ) can improve finish times [9,29], elongate time to exhaustion [14,28,30,37], reduce the gross O_2_ cost of exercise [12,28,30,31,101], and increase peak power [22,29,36] and work rate [12,13,28,36]. With consideration for the above, it should also be noted that other authors have also reported no ergogenic effect of NO_3_^−^ supplementation [32,38,39,40]. Many of these studies have also involved cycling exercise performed by well-trained or elite-level athletes. These will be considered within the remaining sections of this paper, which highlight the use of NO_3_^−^ supplementation to improve exercise performance (e.g., walking, running, arm and leg cycling, rowing, static apnea tests).

### 6.1. Nitrate and Aerobic Performance

NO_3_^−^ supplementation is appreciably neonatal in regards to its development and understanding. In fact, the first study regarding oral supplementation in humans was not published until 2007. Using a cycle ergometer, Larsen *et al*. showed that healthy volunteers—who ingested concentrated NO_3_-water for two 3 day periods—demonstrated lower levels of oxygen uptake (V̇O_2_) at identical cycling workloads when compared to those who ingested the placebo (PL), sodium chloride [41]. Although their findings only displayed lower V̇O_2_ for submaximal cycling work rates, this was one of the first studies to challenge major principles of exercise science regarding dynamics of O_2_ uptake and bioenergetics during exercise.

Before this study, general consensus rested on the idea that oxygen uptake at a given submaximal cycling workload was essentially identical between different people or when measured for the same individual at different occasions. Yet, the authors were able to show that not only could V̇O_2_ be altered for a given submaximal work rate, but that it could be altered through dietary intervention—specifically, using NO3^−^. Most intriguingly, the amount of sodium nitrate supplemented (0.1 mmol NaNO_3_/kg of bw/day) mirrored the amount of NO_3_^−^ found in 150–250 g of NO_3_^−^ rich vegetables. The authors also eliminated the “PL effect” by rearing the sodium chloride and sodium nitrate indistinguishable by taste or appearance.

In 2009, using a double-blind, placebo-controlled crossover study, Bailey and associates [28] supplemented 8 healthy men with 500 mL of BRJ (5.5 mmol of NO_3_^−^/day) or 500 mL blackcurrant cordial (PL) for 6 days to assess V̇O_2_ kinetics in low-intensity and high-intensity cycling exercise. Subjects completed incremental “step” exercise tests on days 4, 5, and 6 starting from a 20-W baseline to moderate and severe-intensity work rates. On day 4, subjects performed two bouts of moderate cycling, while days 5 and 6 were composed of one bout of moderate cycling and one bout of severe cycling. All exercise bouts were 6 min in duration and were separated by a 25 min period of passive recovery; except for day 6, in which the participants continued severe until failure. The authors found an impressive 19% reduction in the amplitude of the pulmonary V̇O_2_ response in the BRJ group, relative to PL, during incremental cycling to the same absolute moderate-intensity work rate [28]. They also found that the gross O_2_ cost of exercise (composed of resting metabolic rate, the O_2_ cost of moving the limbs during baseline pedaling, and the O_2_ cost of muscle contraction to meet the imposed work rate) was ~5% less in the BRJ group [28]. Further, there was a 16% improvement in time to task failure for severe constant work rate cycling in the BRJ group. BRJ ingestion demonstrated to reduce muscle deoxy-hemoglobin amplitude by 13%, suggesting that fractional O_2_ extraction was reduced. In addition, BRJ consumption reduced the amplitude of the V̇O2 slow component (defined as a delayed onset of V̇O_2_ consumption during high intensity exercise). These findings suggest that dietary NO_3_^−^ supplementation results in a reduced gross O_2_ cost of submaximal cycling exercise, and an enhanced tolerance to severe intensity cycling.

A year later, Bailey and colleagues [11] used a randomized, double-blind crossover study involving 7 healthy participants who were required to consume 500 mL of BRJ (5.1 mmol/day of NO_3_^−^) or placebo (NO_3_^−^-depleted) for a period of 6 days. Participants, during the last 3 days of supplementation, then completed low and high (15% and 30% maximal voluntary isometric contractions) intensity step knee extension exercises. The authors noted a 25% increased time to failure for subjects when ingesting BRJ compared to placebo. It was also noted that there was a 25% reduction in the increase in pulmonary V̇o_2_ from rest to low-intensity exercise during the BRJ treatment period. Reductions in phosphocreatine (PCr) degradation were also noted in both low-intensity (~36%) and high-intensity b (~59%) exercise bouts when consuming BRJ [11]. These findings suggest that consumption of NO_3_^−^-rich BRJ may reduce PCr degradation and O_2_ consumption during high and low intensity (knee-extension) exercise. Reductions in PCr degradation also correlated with reduction in total ATP utilization during both high and low intensity bouts of exercise. The authors speculate that the reduced O_2_ observed may correlate to enhanced coupling between ATP hydrolysis and skeletal force production. These effects, along with less accumulation of ADP and Pi, metabolites which are inked to fatigue, seem to enhance exercise tolerance in healthy individuals.

Similarly, in 2010, Larsen performed graded exercise cycle ergometer tests for health volunteers [14]. Subjects ingested 0.1 mmol∙kg^−1^∙day^−1^ of NaNO_3_. O_2_ consumption and metabolic and circulatory parameters were investigated. It was discovered that the metabolic cost of performing standardized constant load exercise was reduced following 3 day consumption of NaNO_3_. It can be inferred from this study that supplementation with NO_3_^−^ may improve metabolic efficiency and a reduced cost of O_2_ for constant-work rate exercise.

Vanhatalo and colleagues studied the effects of acute (1 and 5 days) and chronic (15 days) BRJ consumption on a moderate-intensity exercise bout (90% gas exchange threshold) and an incremental cycle ergometer ramp test (increasing work rate by 1 W every 2 s []) to exhaustion [22]. Cycling exercise performances were scheduled for days 1, 5, and 15 in 8 healthy subjects. Subjects ingested either 500 mL of BRJ (~5.2 mmol of NO_3_^−^) or PL. The study demonstrated that maximal O_2_ consumption (V̇O_2__max_), peak power output, and the work rate associated with the gas exchange threshold were higher following 15 days of BRJ consumption compared to placebo and baseline [22].

In 2011, Lansley and colleagues supplemented 500 mL of NO_3_^−^-depleted BRJ (0.0034 mmol/day of NO_3_^−^) or 500 mL of NO_3_^−^-enriched BRJ (6.2 mmol/ day of NO_3_^−^) [13]. The study used 9 physically active males in a randomized, crossover study design. The subjects reported to the lab on 10 occasions over a 4 to 5 week period. The subjects consumed their assigned treatment for a total of 6 days and were subjected to acute bouts of walking, sub-maximal and high-intensity running (1 bout to exhaustion; 1 bout of 6 min) and incremental knee extension exercises. Subjects completed treadmill testing on days 4 and 5. Knee extension exercises were performed on day 6 for evaluation of mitochondrial oxidative capacity. The authors noted that subjects who consumed the NO_3_^−^-enriched BRJ increased plasma NO_2_^−^ concentration by approximately 105% and reduced O_2_ consumption for moderate constant work rate and high-intensity running by approximately 7% as compared to placebo [13]. The authors also observed that time to exhaustion for 9 athletes who consumed NO_3_^−^-rich BRJ increased during high-intensity running by approximately 15%, while incremental knee extension exercise increased by approximately 5%. Their collective findings suggest that short term (4–6 days) consumption of BRJ may provide performance enhancing benefits during moderate and severe intensity running. Moreover, these findings indicate that the performance benefits are attributable mostly to the high NO_3_^−^ content of BRJ and not its other constituent ingredients. While other nutrients, such as betaine, may prove ergogenic to some degree, the bulk of the effect of BRJ supplementation appears linked to the NO_3_^−^ content.

Then in 2012, Bond and colleagues examined the effects of 6 days of supplementation with BRJ (5.5 mmol of NO_3_^−^/day) in 14 trained, junior male rowers [29]. Using a rowing ergometer, subjects completed 6 maximal 500-m ergometer repetitions and recorded the trial times. The authors indicated improved rowing performance by reporting shortened rowing repetition time, particularly repetitions 4–6 on BRJ (1.7%, 95% CL, ± 1.0%) [29]. These findings suggest that BRJ may specifically enhance exercise tolerance during the latter stages of performance events.

More recently, utilizing a placebo-controlled crossover study, Murphy and colleagues tested eleven healthy and fit men and women in an effort to determine the effect of baked beetroot on running performance [88]. Subjects consumed either baked beetroot (200 g with ≥ 500 mg of NO_3_^−^) or placebo cranberry relish 75 min before a 5 km time trial (TT) treadmill run. The authors noted that individuals consuming the baked beetroot had finishing times approximately 41 s (12.3 ± 2.7 *versusi* 11.9 ± 2.6 km/h, respectively; *p* = 0.06) faster than the placebo condition. During the final 1.8 km of the 5 km run, subjects in the baked beetroot condition had a running velocity 5% faster than those in the placebo condition (12.7 ± 3.0 *versus* 12.1 ± 2.8 km/h, respectively; *p* = 0.02) and rates of perceived exertion were considerably lower than subjects in the placebo condition (13.0 ± 2.1 *versus* 13.7 ± 1.9, respectively; *p* = 0.04). Therefore, NO_3_^−^ consumption may enhance running performance, with specificity to benefits observed during the latter part of the race.

Breese and others investigated the effects of 6 days of supplementation with BRJ on V̇o_2_ kinetics during work-to-work exercise transitions in 9 healthy subjects [30]. Subjects consumed either concentrated BRJ (70 mL 2× day, ~8 mmol of NO_3_^−^) or NO_3_^−^-depleted BRJ (70 mL 2× day, 0.0034 mmol of NO_3_^−^). Utilizing a cycle ergometer, subjects performed a series of progressive cycling load transitions on days 4, 5, and 6 (consecutive) of the supplementation period. Volunteers were first required to cycle for 3 min of unloaded resistance. Immediately after, subjects cycled for 4 min at moderate intensity followed by a final 6 min severe-intensity cycling interval. On the 6th and final day, volunteers transitioned from moderate-intensity cycling to severe-intensity cycling until volitional exhaustion. The authors found that the BRJ group demonstrated enhanced pulmonary V̇o_2_ and muscle deoxyhemoglobin kinetics which lead to increased tolerance for severe-intensity cycling exercise following a transition from a moderate-intensity cycling work rate [30].

The results of the Breese *et al*. study [30] confirm other work indicating that BRJ ingestion may improve exercise performance in simulated competition [12,31] and high intensity intermittent exercise [29,102] It should be noted that most studies involving supplementation of NO_3_^−^ have been performed at constant-work-rate or time to exhaustion tasks. These constant work rate conditions may not sufficiently mirror real-life athletic events and have been criticized for their poor validity and reproducibility [11,28,90]. Future studies may seek to include exercise tasks that more closely mimic real world events.

Similarly, using a double-blind randomized crossover design, Fulford and colleagues tested 8 participants to assess the effects of 15 days dietary NO_3_^−^ on human skeletal muscle metabolism and force production during 50 maximum voluntary contractions (MVC) [35]. The authors found that a significant reduction in the BRJ group’s PCr cost per unit force output at the end of exercise (*p* = 0.04). These results confirm the reduced PCr cost of muscle force production following ingestion of NO_3_^−^-rich BRJ.

### 6.2. Nitrate and Simulated Altitude/Hypoxic Performance

It has been suggested that NO_3_^−^ supplementation can combat a decreased NO production during hypoxic conditions, maintaining or even improving exercise performance. Muggeridge and colleagues recently investigated the effects of a single dose of concentrated BRJ and physiological responses to submaximal exercise and a 16.1-km time trial performance at a moderate simulated altitude (2500 m, ~15% O_2_) [103]. Nine competitive amateur cyclists performed four exercise bouts 3 h post-ingestion of concentrated BRJ (70 mL, ~5 mmol of NO_3_^−^) or a NO_3_^−^-depleted BRJ (70 mL, ~0.01 mmol of NO_3_^−^). The exercise bouts consisted of an initial incremental graded test to exhaustion for determination of peak pulmonary O_2_ uptake and maximum work rate. The second bout consisted of solely baseline performance in hypoxic conditions, while the following two exercise bouts were performed following ingestion of BRJ. The authors found that a single dose of concentrated BRJ reduced the O_2_ cost of steady state cycling exercise and improved 16.1-km time trial performance. These findings suggest that acute (3 h) supplementation with BRJ may provide a simple means of inhibiting the ergolytic effects associated with exercise performance at high altitudes.

Engan and associates recently investigated the effect of dry static apnea performance following NO_3_^−^ supplementation in 12 well-trained apnea divers [34]. Participants were randomly assigned to receive either 70 mL of BRJ (~5 mmol of NO_3_^−^) or 70 mL of PL juice and required to consume 2.5 h prior to apnea tests. Subjects completed two 2-min submaximal apnea performances each separated by a 3-min rest period and followed by a maximal static apnea test. Following acute supplementation, the BRJ group experienced an 11% increase in maximal apneic duration. These findings suggest dietary NO_3_^−^ supplementation as a means for enhancing apneic performance by significantly reducing metabolic costs. 

### 6.3. Summary of Nitrate and Performance

Overall, chronic (3–15 days) and acute (2–3 h) supplementation of nitrate, delivered as either NO_3_^−^-rich BRJ (5.1–18.1 mmol NO_3_^−^ per dose) or NaNO_3_^−^ (0.1 mmol/kg body weight), has demonstrated evidence of reduced pulmonary oxygen uptake correlating to improvements in walking [13,19,28,37], running [13,88], rowing [9,29], and cycling [12,28,30,31,36] submaximal exercise [11,12,22,28,31,37,41] and to improve tolerance at more vigorous work rates [11,12,14,28] across nearly all age groups and in the following populations: untrained males [14], trained males [31,36,38,41,101], well-trained males [12,41], well-trained females [90], healthy men and women [14,22,28,102] and patients with peripheral artery disease [80]. Most studies are now using 70 mL shots of BRJ doses [38,102,103,104].

In this regard, Wylie’s study [102] also recently showed that no improvements to the physiological responses to exercise are obtained with doses of NO_3_^−^ below ~5 mmol. Additionally, it appears that no additional benefit is gained from doses larger than ~8–9 mmol. These findings suggest that doses in the 5–9 mmol range tend to be most beneficial for those seeking improvement to exercise performance.

Acute supplementation (2.5–3 h) with BRJ (~5–6.2 mmol NO_3_^−^, single dose) has also shown ergogenic benefit (e.g., lower VO_2_, enhanced exercise tolerance, increased oxygenated blood levels) in 4 and 16.1 km submaximal cycling time trials [12]; similar benefits have even been noted at simulated altitudes of 2,500 m [103]. Most studies have used mainly young trained males (sample size = ~9) and supplemented 500 mL (~2 cups) of BRJ with varying concentrations of NO_3_^−^ (5–18 mmol NO_3_^−^/day; mostly 5–8 mmol NO_3_^−^/day) and there have been performance enhancements noted mainly for time-to-exhaustion protocols with constant-work-rate exercise (e.g., cycling, running, walking, and step exercises) at submaximal [11,13,28] and high [11,13,28,90] intensities.

Findings for exercise-specific studies involving NO_3_^−^ ingestion are provided in Table 1. It is clear that supplementation of beetroot juice and vegetables rich in inorganic nitrate enhance plasma NO_2_^−^ levels. NO_2_^−^-related NO signaling increases blood flow and targets hypoxic areas of the body. Evidence is now emerging in support of the idea that the NO_3_^−^→NO_2_^−^→NO pathway may play critical roles in human physiology—in particular as related to improving blood flow to help control blood pressure and to aid exercise performance. NO_3_^−^ supplementation (e.g., consumption of particularly BRJ) has demonstrated to reduce blood pressure and the O_2_ cost of submaximal and more severe intensity exercise in humans.

**Table 1:** Studies focused on the impact of nitrate ingestion on exercise performance.

Reference	Subjects	Study Design	Intervention	Exercise Test	Outcomes	Principal Finding
Bailey *et al*. (2010) [11]	7 healthy, active males (age: 28 ± 7)	Randomized, double-blind crossover	500mL of BRJ (5.1 mmol/day NO_3_^−^) for 6 days	Two-legged knee extension ergometer	36% and 59% reduction in the PCr degradation during low and high intensity exercise Reduced muscle ATP turnover rate in both low and high intensity step exercises Improved exercise tolerance of 25% during Two-legged knee-extensor exercise	Significant reduction in the V̇O_2_ cost NO_3_^−^ ingestion doubled plasma NO_2_^−^ levels and reduced SBP (~5 mmHg), DBP (~2 mmHg) and MAP (~2 mmHg)
Bailey *et al*. (2009) [28]	8 healthy men (age: 26 ± 7)	Double-blind, placebo-controlled crossover	500 mL of BRJ (5.5 mmol/day NO_3_^−^) for 4–6 days	Cycle ergometer	Reduced Gross O_2_ cost of exercise (−5%) Increased time to task failure during severe cycle exercise (16%) Decreased amplitude of the pulmonary V̇O_2_ response (19%)	Reduced SBP (~6 mmHg) and the V̇O_2_ slow component Reduced V̇O_2_ cost of sub-maximal cycling and enhanced tolerance to severe intensity cycling Reduced aerobic energy turnover
Bescos *et al*. (2012) [39]	13 male cyclists and triathletes (age: 32.6 ± 5.6)	Double-blind, randomized crossover	Two 3 day periods of BR (10 mg·kg^−1^)	Cycle ergometer	A positive trend between the plasma NO_3_^−^ concentration and increased distance, speed and power in the 40-min distance TT	No significant effect in exercise performance No increase found in plasma nitrated proteins indicating this dose is safe for humans
Bescos *et al*. (2011) [40]	11 male cyclists and triathletes (age: 34.3 ± 4.8)	Double-blind, randomized crossover	Single dose of NaNO_3_ (10 mg·kg^−1^) 3 h before exercise	Cycle ergometer	No effect on exercise performance (time-to-exhaustion) No effect on HR, V̇O_2_, CO_2_	No effect on cardiorespiratory adaptation at low and moderate intensities by a single administration of NaNO_3_ Reduced V̇O_2_ peak in NO_3_^−^ group without affecting maximal attainable work and blood lactate. These findings suggest NO_3_^−^ supplementation may reduce V̇O_2_ peak in well-trained athletes due to increased NO bioavailability
Bond *et al*. (2012) [29]	14 male rowers (age: 16.7 ± 0.5)	Double-blind, randomized crossover	500 mL of BRJ (5.5 mmol of NO_3_^−^) for 5 days	Rowing ergometer	Across all repetitions, performance time was improved with BRJ compared to PL Significant change in DBP in BRJ group compared to PL	Performance benefit data demonstrate that rowing times were improved predominately in the later repetition bouts (4–6)
Breese *et al*. (2013) [30]	9 recreationally active male (*n* = 4) and female (*n* = 5)(age: 30 ± 6)	Double-blind, randomized crossover	BRJ (140 mL/day; ~8 mmol NO_3_^−^) 2 h prior to exercise	Cycle ergometer	Greater average time-to-task failure for severe-intensity cycling exercise in BRJ group (22%) BRJ supplementation appears to enhance phase II V̇O_2_ kinetics during transition from moderate to more severe cycling intensities (lower to higher metabolic rate) BRJ ingestion significantly increased tolerance to severe intensity exercise	Nitrate may induce enhanced matching of O_2_ distribution to contracting skeletal muscle (specifically, type II muscle fibers) BRJ enhances tolerance to severe intensity cycling in recreationally active adults
Cermak *et al*. (2012) [32]	20 well-trained male cyclists (age: 26 ± 1)	Double-blind, repeated measures crossover	Single bolus of BRJ (140 mL; 8.7 mmol NO_3_^−^) 1 h prior to cycling TT	Cycle ergometer	Higher Plasma NO_2_^−^ levels BRJ group No change in TT performance, PO or HR between groups	Ingestion of a single bolus of BRJ, 2.5 h prior to a 1 h cycling TT, did not enhance performance in well-trained cyclists These findings suggest that, in well-trained populations, it may be necessary to increase the NO3^−^ dose or the supplementation period (*i.e.*, >1 day) if seeking ergogenic benefit
Cermak *et al*. (2012) [31]	12 trained male cyclists or triathletes (age: 31 ± 3)	Double-blind, repeated measures crossover	140 mL of concentrated BRJ (~8 mmol NO_3_^−^) for 6 days	Cycle ergometer	Mean V̇O_2_ was lower by 3.5% and 5.1% at 45% and 65% of maximal power with BRJ than with PL Completion of the 10 km TT was 1.2% faster with BRJ than with PL and was associated with a 2% higher mean PO	Ingestion of BRJ for 6 days reduces V̇O_2_ during submaximal cycling exercise in trained athletes BRJ is an effective ergogenic aid for cycling performance in trained adults
Christensen *et al*. (2012) [33]	10 elite male cyclists (age 29 ± 4)	Randomized, single-blind crossover	500 mL of BRJ for 6 days	Cycle ergometer	No effects on V̇O_2_ kinetics, endurance capacity, and repeated sprint performance No difference in peak and mean PO between groups	6 day BRJ supplementation elevated levels of plasma nitrite that did not correspond to changes in V̇O_2_ kinetics, endurance, or repeated sprint performance for elite male cyclists
Engan *et al*. (2012) [34]	12 (9 men, 3 women) trained apneic divers (age: 32 ± 7)	Double-blind, randomized crossover	70 mL of BRJ (~5.0 mmol of NO_3_^−^) 2.5 h prior to apnea testing	A series of two 2-min (sub-maximal) apneic performances 3 min recover intervals followed by a 5 min recovery period before a final maximal effort apneic performance	Acute BRJ consumption increase in maximal apneic duration compared to PL (11%) Higher arterial O_2_ saturation in sub-maximal apneic performances compared to PL	Acute BRJ consumption may enhance apneic performance by reducing metabolic costs for apneic athletes NO_2_^−^ dependent production of NO and accompanying hemodynamic benefits may be pronounced and prolonged in hypoxic conditions
Fulford *et al*. (2013) [35]	8 physically active males (age: 24 ± 4)	Double-blind, randomized crossover	500 mL/d of NO_3_ˉ-rich BRJ for 15 days	One-legged Knee Extension50 Maximal voluntary contractions (MVCʼs) at 2.5 h, 5 day, and 15 day post-intervention	The PCr cost per unit force for the MVC's was reduced in BRJ group compared to PL At the end of the MVC protocol, the mean force to PCr depletion ratio was significantly higher for BRJ group compared to PL	The PCr cost of maximal force production was reduced following BRJ ingestion, indicating a lower PCr cost of force production These findings suggest that chronic supplementation with BRJ enhances contracting skeletal muscle blood flow and improves muscle efficiency
Hoon *et al*. (2014) [105]	10 highly trained male rowers	Double-blind, placebo-controlled crossover	Supplementation with PL or BRJ, a single (4.2 mmol NO_3_^−^), or double (8.4 mmol of NO_3_^−^) dose 2 h prior to rowing exercise	Rowing ergometer 2000 m rowing ergometer TT	Plasma NO_2_^−^ and NO_3_^−^ levels showed evidence of a dose-response effect, with greater amounts of ingested nitrate producing higher plasma NO_2_^−^ levels (DOUBLE > SINGLE > PL)	Double dose (~8.4 mmol of NO3^−^), rather than a Single dose (~4.2 mmol of NO_3_^−^), likely improves 2000 m rowing TT performance when consumed 2 h prior to exercise
Kelly *et al* (2013) [19]	6 male and 6 female older adults (age: 63 ± 2; 64 ± 4)	Randomized, double-blind crossover	3 day supplementation with 140 mL/day (~9.6 mmol /day NO_3_^−^) of BRJ	Treadmill and custom leg ergometer; cognitive function tests; single-leg knee-extension	BRJ ingestion significantly increases plasma [NO_2_^−^] and reduced resting SBP (~5 mmHg), DBP (~4 mmHg) and MAP (~3 mmHg) Magnitude of PCr depletion in low-intensity knee-extension exercise was reduced by ~15% with BRJ group compared to PL, although this difference was not statistically significant	Short-term ingestion of BRJ significantly reduced V̇O_2_ mean response time during treadmill walking BRJ may reduce the risk of hypertension and improve V̇O_2_ kinetics in older adults
Kelly *et al*. (2013) [36]	9 active males (age: 22 ± 3)	Randomized, double-blind crossover	500 mL of BRJ (~8.2 mmol NO_3_^−^/day) for 7–12 days	Cycle ergometer	BRJ improved exercise tolerance by 17%, 16%, and 12% for 60%, 70%, and 80% peak power cycling, respectively BRJ ingestion significantly reduced SBP (~4 mmHg) compared to PL	7–12 day supplementation with BRJ increased severe-intensity cycling exercise tolerance Acute BRJ supplementation is an effective ergogenic aid for cycling exercise for males at the sub-elite level
Kenjale *et al*. (2011) [37]	4 male and 4 female PAD patients (age 67 ± 13) (3 Caucasian, 5 African American)	Randomized, open-label crossover	0.5 L of BRJ (18.1 mmol/L NO_3_^−^) on 2 occasions separated by 7–14 days Note subjects consumed BRJ or PL (orange juice) 3 h prior to CPX	TreadmillMaximal cardiopulmonary exercise (CPX) test	Increased exercise tolerance in patients with peripheral artery disease (walked 18% longer before COT and a 17% longer walking time) Decreased fractional O_2_ extraction (48% decrease in Hgb peak-curve amplitude) at the working tissues during CPX testing compared with PL Decreased DBP at rest and SBP and HR during recovery from maximal exercise	BRJ enhanced circulating levels of [NO_2_^−^] approximately 6-fold; peak concentrations occurred 3 h post BRJ consumption BRJ is associated with a lower O_2_ cost at the working tissues in patients with PAD; Rise in [NO_2_^−^] was associated with an increase in COT and PWT BRJ ingestion as an adjunctive treatment method for ischemic patients
Lansley *et al*. (2011) [12]	9 physically active men (age: 22 ± 4)	Randomized, double-blind crossover	500 mL of BRJ/day (~6.2 mmol of NO_3_^−^) for 6 days	Treadmill Knee-extension	15% and 5% increase in time to task failure during severe intensity running and incremental knee exercise Reduced V̇O_2_ (7%) for constant-work-rate moderate-and severe- intensity running was reduced by 12%–14% reduction in O_2_ cost of walking following NO_3_^−^ supplementation	Reduced O_2_ cost of constant-work-rate moderate-and severe-intensity running Acute (4–6 days) NO_3_^−^ supplementation increases time to task failure for severe intensity running and incremental knee exercise
Lansley *et al*. (2011) [13]	9 club-level competitive male cyclists (age 21 ± 4)	Randomized crossover	500 mL of BRJ (6.2 mmol of NO_3_^−^) or ~2.75 h before completion of a 4 and 16.1 km cycling TT	Cycle ergometer 4.0 and 16.1-km cycling TT’s	Acute dietary NO_3_^−^ supplementation improved 4 and 16.1 km TT performance in competitive cyclists by 2.8% and 2.7% BRJ ingestion resulted in greater cycling PO with no change in V̇O_2_	Plasma [NO_2_^−^] significantly increased 2.5 h postingestion of BRJ Acute (2.5 h) BRJ ingestion may enhance 4 and 16.1 km TT performance in sub-elite cyclists
Larsen *et al*. (2011) [14]	11 male and 3 female (age: 25 ± 1)	Randomized, double-blind crossover	Sodium nitrate (0.1 mmol/kg/day NaNO_3_) for 3 days before testing Note: subjects consumed 0.033 mmol NaNO_3_/kg of body weight 3× daily with last dose taken 90 min before testing	Cycle ergometer	The effective P/O ratio during submaximal ADP stimulation was increased by 19% after NO_3_^−^ supplementation (from 1.36 ± 0.06 to 1.62 ± 0.07), suggesting an improved mitochondrial efficiency Reduction in ANT protein levels (major determinant of mitochondrial proton leak) Increased P/O ratio correlated with reductions in energy expenditure and increases in watt/V̇O_2_	NO_3_ˉ-induced increase in mitochondrial efficiency by reduced leakage/slippage of protons across the inner mitochondrial membrane This is likely due to a reduced expression of ANT protein levels in mitochondria A greater reduction in O_2_ consumption *in vivo* was reflected by increases in mitochondrial efficiency *in vitro* The improvement in exercise efficiency is likely taking place at the mitochondrial level
Larsen *et al*. (2010) [15]	7 male and 2 female (age 30 ± 2.3)	Randomized, double-blind crossover	Sodium nitrate (0.1 mmol/kg/day NaNO_3_) for 2 days before testing Note: subjects consumed 0.033 mmol NaNO_3_/kg of body weight 3× daily with last dose taken 40 min before testing	Arm and leg cycle ergometers	No significant reduction in RBP/SBP Single dose of NO_3_^−^ reduced V̇O_2_ by 80 mL/min Reduced V̇O_2max_ from 3.72 ± 0.33 to 3.62 ± 0.31 L/min) DBP was significantly lower than baseline (~7 mmHg, *P* = 0.04) in NaNO_3_ group 2 min after exercise termination Increased plasma [NO_2_^−^] levels after supplementation period (142 ± 35 nM) compared to PL (61 ± 11 nM)	Reduced O_2_ cost of submaximal arm and leg cycling at work rates expected to require 45%–85% V̇O_2max_ Increase in time to exhaustion following NO_3_^−^ supplementation (despite reduced V̇O_2_) NO_3_^−^ supplementation decreases V̇O_2max_ at maximal combined arm and leg exercises in healthy volunteers
Larsen *et al*. (2007) [41]	9 well-trained men (age: 28 ± 6)	Randomized, double-blind crossover	2 separate 3 day periods of dietary sodium nitrate (0.1 mmol/kg/day NaNO_3_)	Cycle ergometer	Lower O_2_ demand of submaximal cycling exercise Decreased V̇O_2_ over 4 lowest submaximal work rates No difference in lactate concentration, HR, ventilation, or RER between groups during any submaximal work rate	Reduced O_2_ cost of submaximal cycling exercise without an accompanying increase in lactate concentration
Martin *et al*. (2013) [104]	9 male and 7 female team-sport athletes (age: 22.3 ± 2.1; 20.7 ± 1.3)	Randomized, double-blind crossover	70 mL of concentrated BRJ (~5 mmol of NO_3_^−^) 2 h prior to repeated sprint performance	Cycle ergometer Repeated sprint	No difference in mean PO between groups Fewer sprints and less total work in the BRJ group relative to PL	BRJ supplementation is not an effective on repeated sprint performance at near-maximal intensities in young athletes
Masschelein *et al*. (2012) [101]	15 physically active males (age: 21.1 ± 1.0)	Randomized, single-blind crossover	~500 mL (0.07 mmol NO_3_^−^/kg of bw/day) of BRJ for 6 days and 3 h prior to exercise Simulated 5000 m altitude	Cycle ergometer3 EX Trials: 1 trial in normoxia, 2 trials in severe hypoxia (11% ambient O_2_)	In hypoxic conditions, during rest and moderate intensity exercise, arterial O_2_ saturation was 3.5% and 2.7% higher and V̇O_2_ was lower with BRJ *vs*. placebo Reductions in V̇O_2_ max decreased by 5% in hypoxic conditions with BRJ *vs*. placebo	6 day BRJ supplementation improves muscle oxygenation status during both submaximal and maximal exercise in severe acute hypoxia 6 day BRJ supplementation improves arterial and muscle oxygenation statues but not cerebral oxygenation status during exercise in severe hypoxia
Muggeridge *et al*. (2014) [103]	9 competitive amateur male cyclists (age: 28 ± 8)	Double-blind randomized crossover	70 mL of NO_3_^−^-enriched BRJ (~5 mmol of NO_3_^−^) or NO_3_^−^-depleted BRJ (~0.01 mmol of NO3^−^) prior to 2nd and 3rd EX trials	Cycle ergometer; 4 EX trials	A single dose of BRJ lowered V̇O_2_ during submaximal cycling and enhanced TT performance by 2.9% of trained cyclists in normobaric hypoxia V̇O_2_ was significantly lower in the BR trial compared with PLA (*P* = 0.049; 95% CI, 1.3–369.5 mL/min)	Ingestion of BRJ may be a practical and effective ergogenic aid for endurance exercise at altitude Ingestion of BRJ also resulted in a small increase in SpO_2_ compared with the PL condition, although differences did not reach statistical significance
Muggeridge *et al*. (2013) [38]	8 trained male kayakers (age: 31 ± 15)	Randomized crossover	70 mL of BRJ (~5 mmol NO_3_^−^) or placebo 3 h before 2nd and 3rd of 4 performance trials	Kayak ergometer; 4 EX trials	Plasma NO_2_ˉ levels were higher in subjects who ingested BRJ BRJ caused lower V̇O_2_ during submaximal exercise at 60% maximal HR compared to placebo	BRJ ingestion has no effect on repeated supramaximal sprint or 1 km TT kayaking performance Smaller elevation in plasma NO_2_^−^ following single dose of BRJ and individual variability in response to BRJ ingestion may account for findings
Murphy *et al*. (2012) [88]	5 men and 6 women (age: 18 to 55)	Double-blind, placebo-controlled crossover	Baked beetroot (200 g with ≥ 500 mg of NO_3_^−^) 75 min prior to exercise performance	Self-paced 5-km Treadmill TT	Mean running velocity faster after BR consumption compared to PL(12.3 ± 2.7 *vs*. 11.9 ± 2.6 km/h) During last 1.1 miles (1.8 km) of the 5-km run, running velocity was 5% faster in BR group (12.7 ± 3.0 *vs*. 12.1 ± 2.8 km/h)	Whole BR consumption improved 5-km running performance NO_3_^−^-rich whole vegetables also improve exercise capacity
Vanhatalo *et al*. (2011) [22]	moderately trained 7 male and 2 female (age: 28 ± 7)	Randomized, double-blind crossover	750 mL of BRJ (9.3 mmol NO_3_^−^) 24 h prior to the hypoxia trials	Knee-extension ergometer 1× in normoxia (20.9% O_2_; CON) and 2× in hypoxia (14.5% O_2_)	BRJ reduced hypoxic muscle metabolic perturbation (indicated by PCr degradation and Pi accumulation) during high-intensity exercise, and returned exercise tolerance to normoxic conditions PCr recovery kinetics in hypoxic conditions improved by ~16% in BRJ group relative to PL group	Enhanced PCr recovery kinetics in hypoxia due to better NO^.^-mediated matching of tissue O_2_ supply to local metabolic rate BRJ reduced muscle metabolic perturbation during hypoxic exercise and restored exercise tolerance and oxidative function to values of normoxia condition
Vanhatalo *et al*. (2010) [47]	5 male and 3 female (age: 29 ± 6)	Balanced, randomized crossover	500 mL of BRJ (5.2 mmol/day NO_3_^−^) for 15 days	Cycle ergometer 2× moderate-intensity step tests followed by 1× ramp test	Elevated plasma [NO_2_^−^] by 36% at 2.5 h post-ingestion with the highest values attained at 12 (59%) and 15 (46%) days Reduced MAP, DBP, and SBP, V̇O_2_ max, peak PO, and the work rate associated with the anaerobic threshold were higher than the placebo and baseline after 15 days of BRJ consumption	Lower O_2_ cost of moderate-intensity step cycling exercise and these effects are maintained for at least 15 days if supplementation is continued Lower SBP/DBP and elevated [NO_2_^−^] most prominent after 12 days of BRJ supplementation
Wilkerson *et al*. (2012) [106]	8 well-trained male cyclists (age: 31 ± 11)	Randomized, single-blind crossover	500 mL of BRJ (~6.2 mmol NO_3_^−^) 2.5 h before exercise testing	50 mile TT on cycle ergometer	50 mile TT completion was improved by 0.8% No change noted in PO but V̇O_2_ was lower in BRJ group Significant correlation (*r* = −0.83) between increase in plasma [NO_2_^−^] and improvement in TT performance	Acute BRJ ingestion does not significantly alter 50 mile TT performance However, the group mean improvement in completion time of 0.8% may still be meaningful in competition Evidence of “responders” and “non-responders” which may be a function of training status or supplementation regimen
Wylie *et al*. (2013) [102]	10 active men (age: 23 ± 5, S1 age: 22 ± 5, S2)	Balanced crossover	70 mL (4.2 mmol NO_3_^−^), 140 mL (8.4 mmol NO_3_^−^), or 280 mL (16.8 mmol NO_3_^−^) of BRJ 2.5 h before exercise tests	Cycle ergometer Two 5-min bouts of moderate-intensity (93 ± 11 W) and one bout of severe-intensity (258 ± 23 W) until task failure	140 mL and 280 mL intake of BRJ reduced steady-state V̇O_2_ during moderate-intensity exercise by 1.7% and 3.0% and increased time-to-task failure by 14% and 12% Peak reduction in SBP occurred 4 h post-ingestion in all supplementation groups Evidence of “nonresponders” decreased as dose ingestion increased Supplementation with 4.2 mmol of NO_3_^−^ did not enhance time-to-task failure or alter any physiological responses to moderate or severe-intensity exercise relative to PL	Dose-response relationship exists and is important to elicit benefits to exercise tolerance These data suggest no additional ergogenic benefit gained following double dose (8.4 mmol) as compared to triple dose (16.8 mmol) Reduced O_2_ cost of moderate-intensity cycling exercise with supplementation up to 16.8 mmol of NO_3_^−^ (No benefits from single dose)
Wylie *et al*. (2013) [90]	14 active males (age: 22 ± 2)	Double-blind, randomized, crossover	4 × 70 mL of BRJ (~4.1 mmol of NO_3_^−^: 70 mL/day); On each testing day, 2 × 70 mL 2.5 h prior to and 1 × 70 mL 1.5 h prior to exercise protocol	Submaximal and exhaustive Yo-Yo IR1 test	At resting baseline the [NO_2_^−^] was 118 ± 44 nM in PL and 584 ± 343 nM in BRJ Overall, [NO_2_^−^] was greater in BR than PL at each measurement time point and was ~377% greater, on average, across entire protocol During the exhaustive test, however, the [NO_2_^−^] declined by 20 ± 26 nM (20%) in PL and by 288 ± 221 nM (54%) in BR relative to the pre-exercise baseline	Acute dietary nitrate supplementation can improve intermittent high-intensity exercise performance Plasma [NO_2_^−^] was elevated prior to exercise with BRJ compared to PL and declined to a greater extent with BRJ compared to PL during the exhaustive Yo–Yo IR1 test, suggesting that NO_2_^−^ may have served as a substrate for NO production during high-intensity exercise.

With consideration for the above, it should also be noted that not all studies demonstrated a significant performance benefit following NaNO_3_ or BRJ ingestion [32,38,39,40]. For example, a recent study performed by Christensen and colleagues involved 10 elite male cyclists in a randomized, single crossover study [33]. Subjects ingest either 500 mL of BRJ or placebo for a 6 day interval. No increase in V̇O_2_ kinetics or performance was noted. Their findings are supported by work of Cermak and colleagues [32]. Using 20 male trained cyclists, they administered a single bolus of BRJ (140 mL; 8.7 mmol NO_3_^−^) or placebo 2.5 h prior to a 1 h cycling time trial test. Though increases in plasma NO_2_^−^ levels were observed, there were no differences in cyclists’ TT performance, power output, or heart rate between BRJ and PL groups [32].

Several explanations have been offered for the failure of NO_3_^−^ supplementation to elicit an ergogenic effect in the above studies. Some authors [33,38] suggested that the failure to elicit an ergogenic effect may be directly correlated to individual response variability among participants. These findings may suggest that athletes, ranging in status from trained to elite, may require larger doses of NO_3_^−^ or perhaps are more resistant to NO_3_^−^ supplementation. It can also be speculated that there is reduced microbiota reductase activity and/or reductions of incident anaerobes due to variable lifestyle choices among participants. It should also be noted that two [33,38] of these studies supplemented only a single dose of concentrated BRJ which likely lead to smaller elevations in plasma NO_2_^−^ levels. A longer supplementation may prove beneficial. Further, some [39,40] of these studies also showed potential evidence for ergogenic aid of NO_3_^−^ to exercise, however many of these parameters failed to reach levels of statistical significance.

### 6.4. Nitrate and Mechanisms of Actions to Aid Performance

The mechanism by which nitrate acts to enhance muscle metabolic efficiency response, is a topic of debate. It has been proposed that enhanced NO levels (consequent ingestion of nitrate) may attenuate the ATP/PCr cost associated with skeletal muscle force production. Utilizing 31-phosphorous magnetic resonance spectroscopy (^31^P-MRS) for *in vivo* analysis of exercising vastus lateralis muscle, Bailey *et al*. [11] found a significant reduction in muscular accumulation of [Pi] and [ADP] following ingestion of BRJ. Their findings showed a 21% decrease in the [Pi] for maximal voluntary contractions of vastus lateralis muscles during low-intensity exercise. These findings also demonstrated a reduction in depletion of PCr stores following supplementation with BRJ and suggest a reduction in gross ATP cost or turnover for the same work rate.

Larsen and colleagues have also suggested a possible mechanism in which nitrate directly alter mitochondrial efficiency through attenuation of the P:O ratio. [14] It has been hypothesized that a reduction in the gross oxygen cost of exercise is obtained via a reduced proton leakage about the mitochondrial membrane. Further supporting this evidence is the decreased expression of adenine nucleotide translocase which may be responsible for the decrease in proton leakage. To better understand the potential mechanisms of action of NO_3_^−^, they recently studied the impact of dietary NO_3_^−^ on skeletal mitochondria [14]. Following 3 days of NO_3_^−^ supplementation, mitochondria were isolated from vastus lateralis skeletal muscle and displayed almost a 20% improvement in mitochondrial efficiency (as evident by the phosphate to oxygen ratio, P/O) and a 45%–64% decrease in basal O_2_ consumption and state 4 respiration *versus* placebo [14]. Indeed, the P/O ratio is an effective method for energy cost assessment, as it is a measure of the efficiency of coupling of phosphorylation and oxidation in human mitochondria. The P/O demonstrates the amount of phosphates incorporated into ATP energy molecules to the amount of oxygen consumed. In theory, the ratio represents the number of phosphates used to form ATP for every two electrons that are donated from a substrate to each oxygen atom in molecular O_2_. A higher P/O is indicative of higher energy efficiency in that more ATP energy molecules are produced per oxygen atoms reduced to water. This effect correlated with improved metabolic efficiency during exercise, observed in the same volunteers.

## 7. Conclusions

This review focused primarily on the impact of dietary nitrate to aid physical performance and covered 31 studies, inclusive of over 300 participants. Quantitative analysis suggests that performance enhancing benefits are noted predominately with doses ranging from 5 to 9 mmol of NO_3_^−^, delivered as either a single bolus or as multiple (e.g., 1–15) daily servings of said dosage ingestion. This correlates to about 500 mL (~2 cups) of BRJ (~5–9 mmol of NO_3_^−^) at each dosing, with peak elevations in plasma NO_2_^−^ resulting 2.5 and 3 h post-ingestion. These peak plasma NO_2_^−^ concentrations appear to directly correlate with peak reductions in systolic and diastolic blood pressure and may be related to peak physical performance as well. Benefits have been noted with up to 15 days of chronic NO_3_^−^ supplementation and appear to persist 24 h post-ingestion. No additional benefits have been noted by increasing NO_3_^−^ concentration above 8.4 mmol and up to 18.1 mmol of NO_3_^−^, although further research is needed in this area.

Dietary intake is perhaps the most important environmental variable in controlling our overall health. It is particularly relevant to analyze foods on the basis of their constituent ingredients and specifically how these ingredients act (e.g., synergistically, additively, or negatively) within the human body. The interplay of NO_3_^−^ and other nutrients commonly found in vegetables and BRJ (e.g., vitamin C, polyphenols and fatty acids) remains an important area of investigation as these interactions may amplify or repress the health and performance effects associated with NO_3_^−^.

Growing evidence suggests that ingestion of inorganic nitrate—richly found in beetroot juice—enhances blood flow in human and animal microvasculature and leads to reductions in BP, both systolic and diastolic. NO_3_^−^ and the BP benefits following intake also appear to be sex-dependent which may be due differences amongst *in vivo* handling or incident lingual bacteria. Additionally, moderate dietary NO_3_^−^ has demonstrated capacity to impede platelet function, prevent endothelial dysfunction following ischemia-reperfusion and decrease the O_2_ cost associated with exercise. In some cases moderate dietary NO_3_^−^ also appears to enhance exercise tolerance. Indeed, NO_3_^−^ mostly in the form of BRJ and NaNO_3_ has shown remarkable capacity to reduce blood pressure and may provide relief to those experiencing hypertension, hypoxia, and ischemia These findings are paramount to the field of medicine as many patients suffer from blood flow disorders that impair their ability to exercise at low intensities (e.g., walking or standing for long periods). The declining functional capacity that is associated with these cardiovascular disorders greatly impairs an individual’s ability to maintain a satisfactory quality of life. In this regard, ingestion of dietary NO_3_^−^, simply obtained by diets rich in vegetables or by drinking about two cups of BRJ, may provide a low-cost treatment for individuals with blood flow disorders.

As such, individuals suffering from hypertension, peripheral artery disease and ischemia-related diseases (e.g., blood flow disorders) may be relieved by regular consumption of nitrate-rich vegetables—or perhaps using beetroot juice as a source for enhancing blood flow both at rest and during exercise. Individuals seeking said benefits should note that ingestion of 500 mL (~2 cups) of BRJ provides sufficient NO_3_^−^ (~5–9 mmol) content to provide ergogenic and health benefit. Athletes looking to utilize BRJ for ergogenic aid should note that BRJ should be consumed approximately 2.5–3 h prior to an exercise bout. A picture is emerging supporting increased intake of NO_3_^−^-rich natural vegetables and beetroot juice as a low cost and natural method for the prevention and treatment of cardiovascular disease and ischemia-related diseases. These findings are important for cardiovascular medicine and may provide relief to those individuals with impaired blood flow; allowing them to exercise at low-intensities for longer periods of time and providing them with improved quality of life.

Of interest to athletes, health enthusiasts, and physicians alike is the NO_2_^−^/NO demonstrated capacity to elicit several responses in human (and animal) vasculature and skeletal muscle that result in a: (1) reduced gross O_2_ cost of cardiovascular exercise; (2) improved exercise tolerance; (3) reduced ATP cost of force production; (4) improved blood flow and nutrient delivery; and (5) improved revascularization in mice induced with chronic ischemia. This, along with other growing bodies of evidence, suggests a role for the NO_3_^−^→NO_2_^−^→NO pathway in hypoxic tissue (e.g., exercising muscle, ischemic areas) with regards to vasodilation and perfusion.

The available evidence indicates that NO_3_^−^ supplementation for moderate performance improvement in constant-load and incremental-load time trial and time to exhaustion tasks such as walking, running, rowing, and cycling could prove beneficial—for inactive to well-trained individuals and even for patients with PAD. These benefits may prove meaningful in elite sport and ischemia-related diseased contexts. Several authors have reported dose-dependent relationships that are likely related to individual training status, number or type of incident NO_3_^−^-reducing microflora, exercise mode, or supplementation period. It is also important to note that when supplementing NO_3_^−^ in human subjects, it may be of value to determine if these individuals regularly use antibacterial mouthwash or swim in chlorinated pools, as both may potentially inhibit the activity of NO_3_^−^-reducing bacteria, impacting the response to NO_3_^−^ supplementation.

Additional research is needed to determine the impact of NO_3_^−^ supplementation on anaerobic exercise performance—specifically resistance exercise of high volume—as individuals performing such exercise are often the main target market for many dietary supplements containing NO_3_^−^. Studies should also seek to identify the synergistic relationship between isolated NO_3_^−^ and other ingredients found in NO_3_^−^-rich vegetables, to explore the specific dose-response relationships needed to elicit health and ergogenic benefits, and to prolong the supplementation period beyond a relatively short period (*i.e.*, >15 days) to determine if more robust effects can be observed with longer-term treatment. Finally, as more individuals are becoming interested in routine use of NO_3_^−^ supplementation, thoroughly examining the long-term safety profile of NO_3_^−^ supplementation should be considered.
